# Inhibition of cerebrovascular raf activation attenuates cerebral blood flow and prevents upregulation of contractile receptors after subarachnoid hemorrhage

**DOI:** 10.1186/1471-2202-12-107

**Published:** 2011-10-27

**Authors:** Saema Ansar, Aida Maddahi, Lars Edvinsson

**Affiliations:** 1 Department of Clinical Sciences, Division of Experimental Vascular Research, Lund University, Sweden; 2 Department of Clinical Experimental Research, Research Park Glostrup, Copenhagen University, Glostrup, Denmark

## Abstract

**Background:**

Late cerebral ischemia carries high morbidity and mortality after subarachnoid hemorrhage (SAH) due to reduced cerebral blood flow (CBF) and the subsequent cerebral ischemia which is associated with upregulation of contractile receptors in the vascular smooth muscle cells (SMC) via activation of mitogen-activated protein kinase (MAPK) of the extracellular signal-regulated kinase (ERK)1/2 signal pathway. We hypothesize that SAH initiates cerebrovascular ERK1/2 activation, resulting in receptor upregulation. The raf inhibitor will inhibit the molecular events upstream ERK1/2 and may provide a therapeutic window for treatment of cerebral ischemia after SAH.

**Results:**

Here we demonstrate that SAH increases the phosphorylation level of ERK1/2 in cerebral vessels and reduces the neurology score in rats in additional with the CBF measured by an autoradiographic method. The intracisternal administration of SB-386023-b, a specific inhibitor of raf, given 6 h after SAH, aborts the receptor changes and protects the brain from the development of late cerebral ischemia at 48 h. This is accompanied by reduced phosphorylation of ERK1/2 in cerebrovascular SMC. SAH per se enhances contractile responses to endothelin-1 (ET-1), 5-carboxamidotryptamine (5-CT) and angiotensin II (Ang II), upregulates ET_B_, 5-HT_1B _and AT_1 _receptor mRNA and protein levels. Treatment with SB-386023-b given as late as at 6 h but not at 12 h after the SAH significantly decreased the receptor upregulation, the reduction in CBF and the neurology score.

**Conclusion:**

These results provide evidence for a role of the ERK1/2 pathway in regulation of expression of cerebrovascular SMC receptors. It is suggested that raf inhibition may reduce late cerebral ischemia after SAH and provides a realistic time window for therapy.

## Background

The clinical syndrome of delayed cerebral ischemia after rupture of a cerebral aneurysm includes recurrent bleeding from the aneurysm, angiographic evidence of cerebral arterial constriction, ischemic deterioration and is associated with high morbidity. Early surgery or angiographic coiling stops the bleeding but still carries high ischemic morbidity; on the other hand late surgery has lower ischemic morbidity but a higher overall mortality, which makes the choice of treatment difficult. Over 300 pharmaceutical agents have been used in unsuccessful attempts to reverse the cerebral vascular narrowing that can be seen after subarachnoid hemorrhage (SAH) (also referred to as vasospasm) and to improve outcome of the patients [1]. Current treatment consists of neurocritical care, measures to prevent and minimize secondary brain injury, calcium channel blockers, and hemodynamic management and endovascular therapies. These manoeuvres are however expensive, time-consuming and only partly effective [2]. The search continues for agents that will prevent or alleviate the cerebral ischemia after SAH.

Several theories have appeared to explain the mechanisms responsible for the late cerebral ischemia after SAH, e.g. enhanced levels of free radicals [3-5], central nervous system dysfunction [6,7], reduced levels of endothelial relaxing factors [8-10], increased levels of inflammatory mediators [11] and increased amounts of vasoconstrictor substances such as endothelin (ET) [12] and 5-hydroxytryptamine (5-HT) [13,14].

We have recently suggested that many of these mechanisms are inter related and may share a common signal-transduction pathway. SAH may cause enhanced expression of endothelin type B receptor (ET_B_), 5-hydroxytryptaimine type 1B receptor (5-HT_1B_) and angiotensin type 1 (AT_1_) receptors, and of genes for cytokines and metalloproteinases [15]. These genes are transcribed via activation of mitogen-activated protein kinases (MAPKs), in particular of the extracellular signal-regulated 1/2 (ERK1/2) kinase pathway that acts via specific transcription factors to result in their protein expression [16]. We and others have shown that the upstream MEK1/2 inhibitor U0126 can reduce the ERK1/2 activity and the infarct volume after middle cerebral artery occlusion (MCAO) in rat [17,18]. Raf is active upstream of MEK and acts specifically to regulate the MEK/ERK1/2 pathway. In experimental studies we have reported that the raf inhibitor SB386023-b effectively blocks pERK1/2 expression and attenuates the cerebrovascular receptor upregulation both on functional and molecular levels [19].

Here we suggest that administration of the specific and potent raf inhibitor SB386023-b prevents contractile receptor upregulation and the development of late cerebral ischemia. The selective and potent raf inhibitor SB386023-b has been demonstrated to inhibit both c-Raf and B-Raf at 1-10 μM in a variety of cellular assays, without affecting Jun N-terminal Kinase (JNK) or p38 [20]. We suggest that the late cerebral ischemia and the cerebral blood flow (CBF) reduction are the result of upregulation of receptors in the vascular smooth muscle cells (SMC) that occur via activation of the ERK1/2 pathway. We suggest as a hypothesis that SB386023-b, given at 0 and 6 h after the SAH improves the neurology outcome, normalizes regional CBF and cerebrovascular receptor upregulation.

## Results

### SAH model

SAH was induced by injecting 250 μl blood into the prechiasmatic cistern in the rat. The raf inhibitor SB-386023-b was injected intracisternally in our rat model at 0, 6, or 12 hours after the SAH.

The total number of rats used in the study was 71; 12 in the sham group, 15 in the SAH + vehicle group, 9 in the SAH group and 35 was used in the SAH + treatment with SB386023-b groups. The mortality rate was 8% and the animals died during the follow up, there was no difference in the mortality rate between the groups. The rats did not show any distressed behaviour. They were moving around normally, eating and drinking. All surviving animals were neurologically examined using an established scoring system [21,22] (Table 1). All SAH + vehicle animals and SAH animals treated with SB386023-b after 12 h received a score of 1, and the sham animals and SAH animals treated with SB386023-b after 0 and 6 h got a score of 0.

**Table 1:** Neurological score after SAH

Score	Interpretation
0	No visible deficits

1	Contralateral forelimb flexion, when hold by tail

2	Decreased grip of contralateral forelimb

3	Spontaneous movement in all directions, but contralateral circling if pulled by tail

4	Spontaneous contralateral circling

5	Death

In all operated rats, mean arterial blood pressure (105 ± 3 mmHg), partial pCO_2 _(38 ± 3 mmHg), partial pO_2 _(108 ± 4 mmHg), hematocrit (39 ± 1 mmHg) values and temperature were within acceptable limits during the operation. No statistical difference was seen in physiological parameters between the groups; sham, SAH + vehicle (henceforth only mentioned as SAH) and SAH treated with SB386023-b at the different time points. As a result of injecting the blood the cortical blood flow dropped over both hemispheres to 10 ± 5% of resting flow (there was no difference between the two Laser Doppler probe data) and the intracranial pressure (ICP) increased from 9 ± 2 to 126 ± 9 mmHg. The Laser Doppler blood flow and the elevated ICP returned to the basal values within one hour of postoperative monitoring. There was no difference between the SAH groups.

### Acute effects of the raf inhibitor SB386023-b on CBF, ICP and functional responses

The acute effects of the raf inhibitor SB386023-b on CBF, ICP and functional responses was investigated.

**Figure 1:**
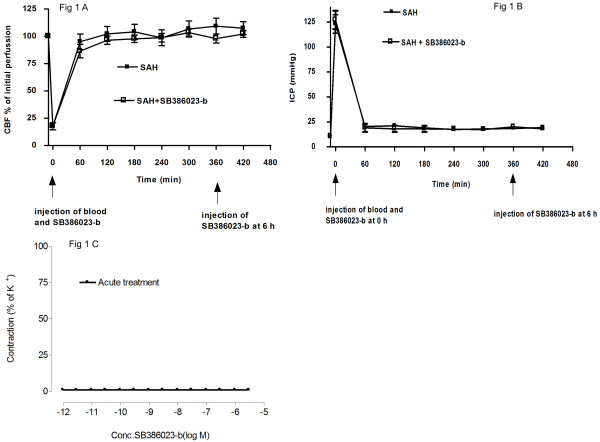
**Illustration of the cortical blood flow measured with laser Doppler flowmetry (A) and ICP (B) at the time of administration of SB386023-b and injection of blood**. (**C**) Concentration response curves elicited by cumulative application of SB386023-b in rat cerebral arteries.

There were no immediate changes in the cortical CBF or the ICP (within the first 2-3 h) when SB386023-b was administrated at 0 h or 6 h after the SAH (Figure 1A-B). In addition, there were no difference in the local cortical blood flow response and ICP during the acute phase between the groups SAH and SAH treated with SB386023-b. This shows that the raf inhibitor SB386023-b has no acute effect on the cortical CBF and ICP.

To study if the raf inhibitor has a direct vasomotor effect on cerebral blood vessels, isolated ring segments of the MCA were studied in a myograph. The functional data shows that SB386023-b had no effect on the contractility when it was applied in increasing concentrations directly on the isolated MCA (Figure 1C). In artery segments precontracted with 5-HT, SB386023-b tended to relax the MCA slightly but the effect was not significant at any concentration (at 10^-5 ^M the relaxant effect was 3 ± 2%).

### Regional cerebral blood flow (rCBF) to evaluate the overall consequences of SAH

The regional and global CBF was investigated by an autoradiographic method in the various groups; sham, SAH and SAH treated with SB386023-b.

**Figure 2:**
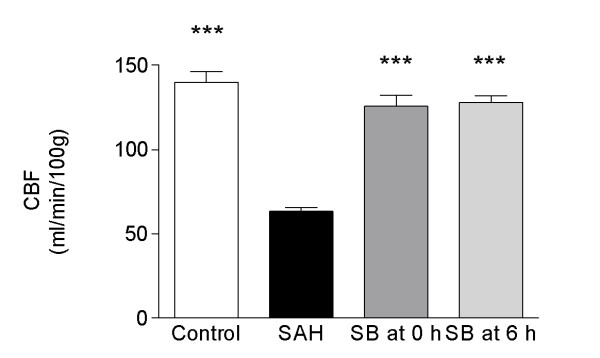
**Effect of treatment with the raf inhibitor SB386023-b on the global CBF after induced SAH in rats**. There is a reduction in the global CBF in the SAH compared to the control rats. Treatment with SB386023-b inhibited this reduction in CBF. Data were obtained by an autoradiographic method and data are expressed as mean ± s.e.m. values, *** P < 0.001. Statistical analyses were performed using Kruskal-Wallis non-parametric test together with Dunn's post-hoc test.

There was a significant global decrease in cerebral blood flow measured at 48 h in the SAH (n = 6) group as compared to the control sham group (n = 6) from 140 ± 6 to 63 ± 2 ml/100 g/min. Treatment with SB386023-b, starting at 0 h and 6 h after the SAH, prevented the reduction in CBF seen after SAH (Figure 2) but not at 12 h (110 ± 20 ml/100 g/min, n = 3). The SAH animals showed a reduction in the regional CBF in 15 of the 18 brain regions examined compared to the control operated rats (Table 2). Treatment with SB386023-b in conjunction with the SAH at 0 and after 6 h of SAH prevented this reduction in rCBF and there was no difference as compared to the control group for any of the regions studied. Treatment with SB386023-b administered at 12 h after induction of SAH did not prevent this reduction in rCBF (data not shown).

**Table 2:** Regional cerebral blood flow 48 hours post subarachnoid hemorrhage

	Control	SAH	SAH + SB386023-b after 0 hours	SAH + SB386023-b after 6 hours
frontal cortex	141 ± 16^a^	75 ± 20^a, b^	132 ± 17^b^	135 ± 19^b^

parietal cortex	169 ± 30^a^	68 ± 14^a, b^	141 ± 28^b^	132 ± 30^b^

occiptal cortex	137 ± 17^a^	67 ± 21^a, b^	141 ± 20^b^	155 ± 21^b^

caudate putamen	131 ± 17^a^	71 ± 20^a, b^	129 ± 16^b^	142 ± 18^b^

Septum	115 ± 24^a^	60 ± 20^a, b^	104 ± 18	128 ± 20^b^

hippocampus	147 ± 25^a^	52 ± 11^a, b^	88 ± 13^b^	85 ± 20^b^

Thalamus	120 ± 16^a^	64 ± 16^a, b^	124 ± 17^b^	136 ± 15^b^

Hypothalamus	107 ± 20	55 ± 16^b^	95 ± 9	127 ± 15^b^

Corpus callosum	80 ± 19	41 ± 18	57 ± 14	87 ± 14

sensorimotor cortex	164 ± 27	74 ± 20	134 ± 15^b^	140 ± 17^b^

superiour colliculus	197 ± 27^a^	70 ± 20^a, b^	121 ± 12^b^	130 ± 17^b^

inferior colliculus	122 ± 5^a^	71 ± 19^a, b^	134 ± 21^b^	67 ± 21^b^

cerebellar cortex	127 ± 25^a^	53 ± 14^a, b^	116 ± 25^b^	135 ± 27^b^

dentate nucleus	133 ± 20^a^	53 ± 14^a, b^	127 ± 17^b^	114 ± 20^b^

facial nucleus	170 ± 31^a^	70 ± 21^a, b^	156 ± 32^b^	131 ± 30^b^

cochlear nucleus	150 ± 30^a^	54 ± 15^a, b^	150 ± 21^b^	119 ± 25^b^

vestibular nucleus	153 ± 18^a^	75 ± 23^a, b^	167 ± 22^b^	130 ± 20^b^

trigeminal nucleus	154 ± 22^a^	65 ± 19^a, b^	149 ± 22^b^	124 ± 23^b^

Values are expressed in ml/min/100 g and given as mean ± s.e.m. ^a ^= significant difference between SAH and control groups, ^b ^= significant difference between SAH and SAH treated with SB386023-b 0 hr and 6 hr post SAH.

### Functional in vitro pharmacology

K^+ ^-induced contractions did not differ significantly between the cerebral arteries from the different groups (Table 3 and 4). The E_max _and pEC_50 _values for respective groups are presented in Table 3 and 4.

**Table 3:** Contractile effects of ET-1 and 5-CT in MCA and basilar arteries

			Biphasic curve				Sigmoidal curve	
	**N**	**K^+ ^mean ±** **s.e.m**	**E_max 1_(%) ±** **s.e.m**	**E_max 2_(%) ± s.e.m**	**pEC_50(1) _±** **s.e.m**	**pEC_50(2) _±** **s.e.m**	**E_max _(%) ±** **s.e.m**	**pEC_50 _±** **s.e.m**

**ET-1 MCA**								

Sham	6	1.76 ± 0.57					152 ± 11	8.59 ± 0.14

SAH	7	1.95 ± 0.34	28 ± 8^a, b^	148 ± 5	12.62 ± 0.23^a, b^	9.35 ± 0.22		

SAH+SB386023-b at 0 h	5	2.22 ± 0, 35					133 ± 5	8.87 ± 0, 15

SAH+SB386023-b after 6 h	9	1.81 ± 0, 27					176 ± 6	8.92 ± 0.13

SAH+SB386023-b after 12 h	5	1.45 ± 0.27	27 ± 7^a, b^	149 ± 17	12.02 ± 0.39^a, b^	9.13 ± 0.17		

**ET-1 BA**								

Sham	6	4.00 ± 0.58					119 ± 9	9.37 ± 0.10

SAH	6	3.70 ± 0.47	34 ± 5^a, b^	126 ± 8	12.21 ± 0.15^a, b^	9.67 ± 0.02		

SAH+SB386023-b at 0 h	6	3.92 ± 0.45					124 ± 5	9.23 ± 0.16

SAH+SB386023-b after 6 h	6	4.23 ± 0.41					155 ± 10	8.80 ± 0.13

SAH+SB386023-b after 12 h	6	3.75 ± 0.32	29 ± 5	125 ± 5	12.41 ± 0.18	9.75 ± 0.23		

**5-CT MCA**								

Sham	6	1.67 ± 0.54	17 ± 6^a^	42 ± 9^a^	8.40 ± 0.14	6.08 ± 0.64^a^		

SAH	6	2.35 ± 0.40	35 ± 2^a, b^	60 ± 3^a, b^	8.47 ± 0.17	7.11 ± 0.01^a, b^		

SAH+SB386023-b at 0 h	5	2.45 ± 0.25	16 ± 5^b^	43 ± 8	8.48 ± 0.13	6.39 ± 0.33		

SAH+SB386023-b after 6 h	6	1.81 ± 0.27	21 ± 5^b^	45 ± 17	8.43 ± 0, 11	6.33 ± 0.51		

SAH+SB386023-b after 12 h	6	2.09 ± 0.35	50 ± 6	92 ± 9	8.37 ± 0.10	6.47 ± 0.02		

**5-CT BA**								

Sham	6	3.90 ± 0.62	18 ± 6^a^	56 ± 9	8.00 ± 0.11^a^	6.25 ± 0.15		

SAH	8	4.00 ± 0.26	43 ± 6^a, b^	64 ± 5	8.46 ± 0.21^a, b^	6.68 ± 0.01		

SAH+SB386023-b at 0 h	6	3, 89 ± 0.22	20 ± 4^b^	47 ± 4	8.09 ± 0.21^a^	6.12 ± 0.12		

SAH+SB386023-b after 6 h	6	4.23 ± 0.41	12 ± 3^b^	47 ± 9	7.92 ± 0.23^b^	6.05 ± 0.44		

SAH+SB386023-b after 12 h	6	3.75 ± 0.43	38 ± 5^b^	55 ± 4	8.30 ± 0.19^a, b^	6.48 ± 0.10		

Responses were characterized by E_max _values, expressed as percent of 63 mM K^+ ^-induced contraction, and pEC_50 _values (negative logarithm of the molar concentration that produces half maximum contraction). Values are represented as mean ± s.e.m and n represents number of animal. ^a ^= significant difference between SAH and control groups, ^b ^= significant difference between SAH and SAH treated with SB386023-b

**Table 4:** Contractile effects of Ang II in MCA

	N	K^+ ^mean ± s.e.m	E_max _(%) ± s.e.m	pEC_50 _± s.e.m
**Ang II after PD123319 MCA**				
Sham	6	2.34 ± 0.38	3 ± 2^a^	
SAH	6	1.33 ± 0.28	53 ± 12^a,b^	7.95 ± 0.38
SAH + SB386023-b at 0 h SAH + SB386023-b after 6 h	6 6	1, 74 ± 0.11 1.64 ± 0.12	11 ± 4^b^ 13 ± 5^b^	
SAH + SB386023-b after 12 h	6	1.47 ± 0.54	35 ± 5	8, 69 ± 0.14

Responses were characterized by E_max _values, expressed as percent of 63 mM K^+ ^-induced contraction, and pEC_50 _values (negative logarithm of the molar concentration that produces half maximum contraction). Values are represented as mean ± s.e.m and n represents number of animal. ^a ^= significant difference between SAH and control groups, ^b ^= significant difference between SAH and SAH treated with SB386023-b

#### Contractile response to ET-1

**Figure 3:**
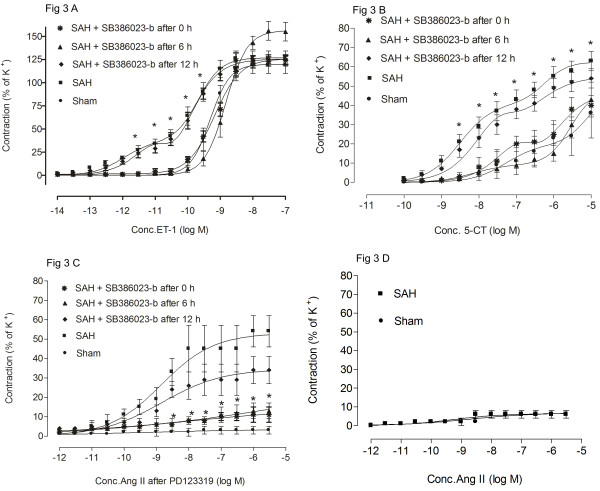
**Concentration response curves elicited by cumulative application of ET-1, 5-CT and Ang II in rat cerebral arteries**. (A) ET-1; BA, (B) 5-CT; BA, (C) Ang II pretreated with PD123319; MCA and (D) Ang II; MCA. Effect of induced experimental SAH, SAH treated with raf inhibitor SB386023-b at 0 h, 6 h and 12 h after SAH and sham operated rats are illustrated. The responses to ET-1, 5-CT and Ang II (pretreated with PD123319) are clearly increased in the SAH compared to sham operated rats. The effects of SAH are clearly inhibited with use of SB386023-b after 0 and 6 hour. Data are expressed as mean ± s.e.m, * P ≤ 0.05. * significantly difference between SAH and SAH treated with SB386023-b at 0 and 6 h. Statistical analyses were performed using Kruskal-Wallis non-parametric test together with Dunn's post-hoc test.

In the middle cerebral artery (MCA) and basilar artery (BA) from SAH rats (n = 6) ET-1 showed a leftward shift of the concentration-response curve which indicates an enhanced contractile response to ET-1 as compared to the sham-operated rats (n = 6) where a sigmoid curve was obtained (Figure 3A, Table 3). Treatment with SB386023-b starting at 0 and 6 h after SAH produced a significantly attenuated ET-1 induced response, compared to the rats with induced SAH. Interestingly there was no significant difference in the contractile response between sham and SB386023-b given at 0 and 6 h after SAH (Figure 3A, Table 3). When the SB386023-b treatment was begun at 12 h after the induced SAH the responses did not differ from that seen in animals receiving only SAH (Table 3).

#### Contractile response to 5-CT

5-CT gave rise to a biphasic concentration-dependent contraction, indicating the presence of the two 5-HT receptor subtypes 5-HT_1B _and 5-HT_2A _as verified by previous detailed antagonist studies [23]. This has been confirmed using GR 55562, a selective 5-HT_1B _receptor antagonist, shifting the high-affinity phase to the right and removing the 5-HT_1B _component of the low-affinity phase [24].

In both MCA and BA from rats with induced SAH (n = 5-6) 5-CT gave rise to an elevated Emax_(1)_, Emax_(2) _and pEC_50(2) _as compared to the sham-operated rats (n = 5-6) (p < 0.05, Figure 3B; Table 3). In BA treatment in vivo with SB386023-b starting at 0 h and 6 h after SAH showed down regulated responses, both the first 5-HT_1B _and the second 5-HT_2A _phases were lower as compared to rats with induced SAH (Figure 3B). In the MCA treatment with SB386023-b at 0 h and 6 h after the SAH showed significantly reduced Emax_(1) _(p < 0.05) and tended to a decrease in the Emax_(2)_, pEC_50(1) _and pEC_50(2) _as compared to SAH (Table 3). SB386023-b treatment given 12 h after the induced SAH did not show attenuated contractile response as compared to SAH (Figure 3B, Table 3).

#### Contractile response to Ang II

In MCA from rats with induced SAH (n = 6) Ang II (acting via AT_1 _receptors) induced a concentration-dependent contraction (in the presence of the AT_2 _receptor antagonist PD123319). Treatment with SB386023-b given at 0 and 6 h after the SAH produced a significantly attenuated Ang II induced response, compared to the rats with induced SAH. Interestingly there was no significant difference in the contractile response between sham and SB386023-b given at 0 and 6 h after the SAH (Figure 3C; Table 4). However, SB386023-b treatment given 12 h after the induced SAH did not show attenuated contractile response as compared to SAH. Ang II did not induce increased contractility in the BA after SAH. In the absence of the AT_2 _receptor antagonist PD123319 there was no increased contractile response to Ang II after SAH as compared to sham (Figure 3D).

### Quantitative mRNA expression

To quantify mRNA for the ET_A_, ET_B_, AT_1_, AT_2 _and 5-HT_1B _receptors, RT-PCR and real-time detection monitoring the PCR products was employed.

**Figure 4:**
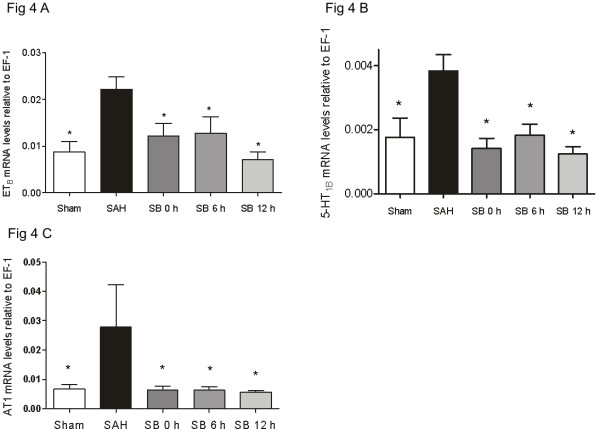
**Effect of treatment with the raf inhibitor SB386023-b in cerebral arteries on the mRNA levels of ET_B_, 5-HT_1B _and AT_1 _receptors after experimental induced SAH in rats**. (A) ET_B_, (B) 5-HT_1B _and (C) AT_1_. There are upregulations of the ET_B_, 5-HT_1B _and AT_1 _receptor mRNA levels in the SAH compared to the sham operated rats. Treatment with SB386023-b prevented the upregulations. Data were obtained by real-time PCR and are expressed as mean ± s.e.m. values relative to EF-1 mRNA levels, n = 7-10, * P ≤ 0.05. Statistical analyses were performed using Kruskal-Wallis non-parametric test together with Dunn's post-hoc test.

The standard curves for each primer pair had almost similar slopes, demonstrating that EF-1, ET_A_, ET_B_, 5-HT_1B_, AT_1 _and AT_2 _cDNA were amplified with the same efficiency (data not shown). In each PCR experiment, a no template control was included, and there were no signs of contaminating nucleic acids in the samples. Since the results from the different brain arteries examined MCA, BA and circle of Willis (n = 7-10) were identical, they were grouped together in the statistical analysis. The results showed that treatment with SB386023-b inhibited the enhanced expression of ET_B_, 5-HT_1B _and AT_1 _receptor mRNA levels significantly as compared to control (Figure 4A-C). There was no difference in the expression of ET_A _and AT_2 _receptor mRNA levels between the three groups sham, SAH and SAH treated with SB386023-b (data not shown).

### pERK1/2 expression examined by Western Blot

The phosphorylated ERK1/2 protein levels was investigated by Western Blot.

**Figure 5:**
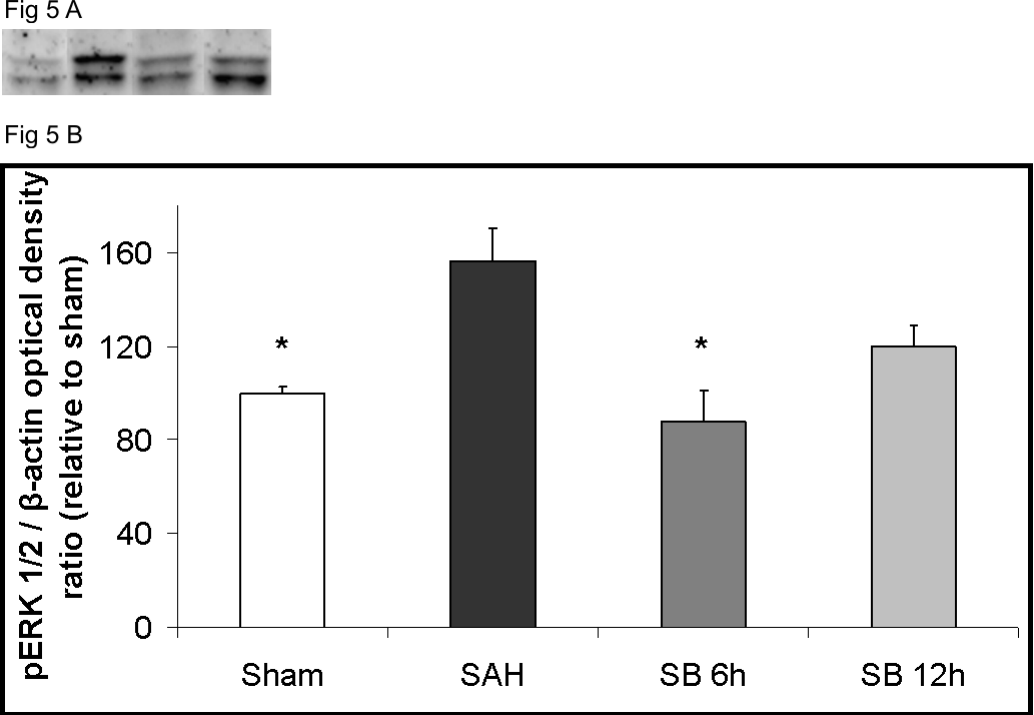
**Effect of treatment with the raf inhibitor SB386023-b in cerebral arteries on the protein levels of pERK1/2 after SAH**. There are increased expressions of the pERK1/2 protein levels in the SAH compared to the sham operated rats. Treatment with SB386023-b after 6 h prevented the increased protein expression. Data are presented as the pERK1/2/β-actin mean optical density ratio relative to control. Data are expressed as mean ± s.e.m. * P ≤ 0.05.

The pERK1/2 protein levels were activated after SAH (156 ± 14%) as compared to sham (100 ± 3%) The treatment with the raf inhibitor SB386023-b at 6 h (88 ± 13%) after SAH prevented the pERK1/2 protein level activation (Figure 5A-B). However, SB386023-b given 12 h after SAH did not attenuate the pERK1/2 protein levels (120 ± 9%) (Figure 5A-B).

### Protein expression examined with immunohistochemistry

**Figure 6:**
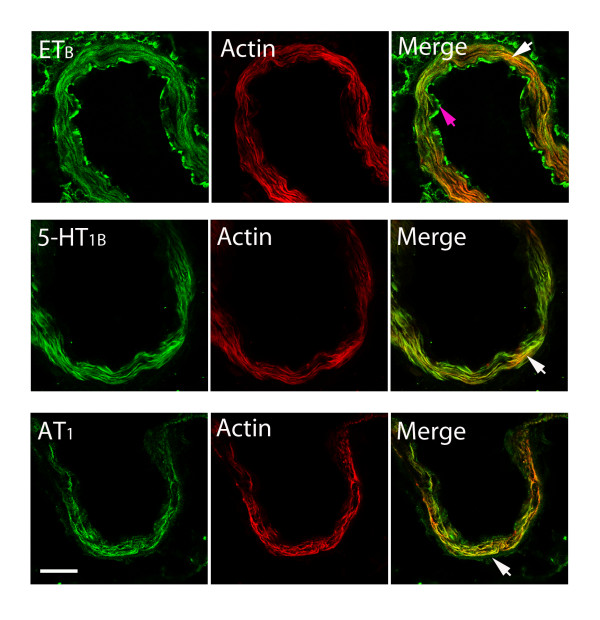
**Double immunofluorescence staining for ET_B_, 5-HT_1B _and AT_1 _and actin in smooth muscle cells of the basilar artery after SAH**. Photographs demonstrating the localization of ET_B_, 5-HT_1B_, AT_1_, actin immunostaining, and their co-localization in smooth muscle cells (yellow fluorescence in the merged picture). Scale bar, 50 μm.

**Figure 7:**
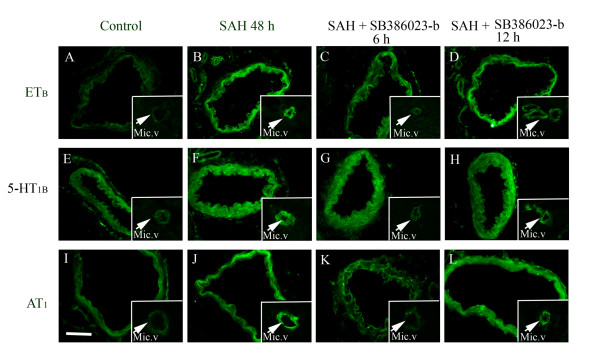
**Sections from the basilar artery showing ET_B_, 5-HT_1B _and AT_1 _immunoreactivity in the smooth muscle cell layer**. a) ET_B_; sham, b) ET_B_; SAH, c) ET_B_; SAH treated with SB386023-b after 6 h, d) ET_B_; SAH treated with SB386023-b after 12 h, e) 5-HT_1B_; sham, f) 5-HT_1B_; SAH, g) 5-HT_1B_; SAH treated with SB386023-b after 6 h, h) 5-HT_1B_; SAH treated with SB386023-b after 12 h, i) AT_1_; sham, j) AT_1_; SAH, k) AT_1_; SAH treated with SB386023-b after 6 h, l) AT_1_; SAH treated with SB386023-b after 12 h. There are increased expressions of the ET_B_, 5-HT_1B _and AT_1 _receptor protein levels in the SAH compared to the sham operated rats. Treatment with SB386023-b after 6 h prevented the increased protein expression in the smooth muscle cells. Data were obtained with confocal microscopy.

**Figure 8:**
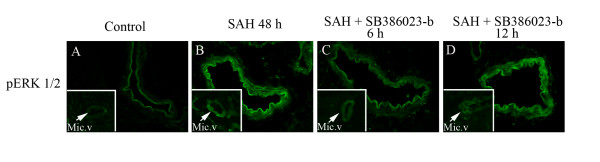
**Sections from the basilar artery showing pERK immunoreactivity in the smooth muscle cell layer**. a) pERK1/2; sham, b) pERK1/2; SAH, c) pERK; SAH treated with SB386023-b after 6 h, d) pERK; SAH treated with SB386023-b after 12 h, There are increased expressions of the pERK1/2 protein levels in the SAH compared to the sham operated rats. Treatment with SB386023-b after 6 h prevented the increased protein expression in the smooth muscle cells. Data were obtained with confocal microscopy.

The localization and activation of the protein levels was examined by confocal microscopy and immunocytochemistry using selective antibodies towards the phosphorylated ERK1/2, ET_B_, 5-HT_1B _and AT_1 _receptors. The results demonstrated that the pERK1/2, ET_B_, 5-HT_1B _and AT_1 _receptors were all present in the cytoplasm of the cerebrovascular smooth muscle cells. Double immunohistochemistry staining versus smooth muscle actin, revealed their co-expression in the smooth muscle cells were performed to verify the localization and in the circle of Willis arteries, the MCA and the BA, the microvessels in the brain (Figure 6). The ET_B _receptor protein was expressed in the smooth muscle cells and this signal was increased in SAH (167 ± 4%) as compared to sham (100 ± 3%). Similarly the 5-HT_1B _(180 ± 2%) and AT_1 _(168 ± 7%) receptor proteins were expressed more in SAH as compared to sham (100 ± 7%) and (100 ± 7%); respectively (p < 0.05 for all). Treatment with the raf inhibitor SB386023-b, starting with administration at 0 h or 6 h after SAH blunted the SAH induced upregulation of ET_B _(109 ± 5%), 5-HT_1B _(121 ± 23%) and AT_1 _(105 ± 10%) receptor protein levels in the smooth muscle cells (Figure 7A-L, Table 5). However, when the SB386023-b treatment was started 12 h after the induced SAH it did not attenuate the upregulated 5-HT_1B _(172 ± 25%) and AT_1 _(180 ± 15%) receptor protein levels in the smooth muscle cell layer as compared to the SAH (Figure 7A-L, Table 5). After SAH the pERK1/2 level (188 ± 7%) was increased in the smooth muscle cells as compared to sham (100 ± 3%). Treatment with the ERK1/2 inhibitor at 0 h and 6 h after starting the SAH prevented the pERK1/2 (102 ± 5%) activation (Figure 8, Table 5). SB386023-b given 12 h after SAH did not attenuate the pERK1/2 (167 ± 9%) (Figure 8, Table 5).

**Table 5:** Activation of the different protein levels measured with immunohistochemistry in basilar artery after SAH

	Sham	SAH	SAH + SB386023-b at 0 h	SAH + SB386023-b after 6 h	SAH + SB386023-b after 12 h
ET_B_ (%) ± s.e.m	100 ± 3	167 ± 4	107 ± 4	109 ± 5	93 ± 4

5-HT_1B_ (%) ± s.e.m	100 ± 7	180 ± 2	124 ± 25	121 ± 23	172 ± 25

AT_1_ (%) ± s.e.m	100 ± 7	168 ± 7	109 ± 8	105 ± 10	180 ± 15

pERK1/2 (%) ± s.e.m	100 ± 3	188 ± 7	101 ± 3	102 ± 5	167 ± 9

Values are expressed as percentage of control and given as mean ± s.e.m.

In addition, as can be seen in Figure 7 and 8, the upregulation was not confined only to the large cerebral arteries but notable also in the brain parenchyma micro vessels but not in the brain tissue proper, in neurons or glial cells. Treatment with SB386023-b (0 and 6 h but not 12 h) reduced also the microvessels receptor expression and the pERK1/2 in the smooth muscle cells.

## Discussion

This study demonstrates that there is a clear association between cerebrovascular receptor upregulation via transcription involving activation of ERK1/2 and the subsequent reduction in CBF after SAH. Specific blockade of the MAPK ERK1/2 activity with a raf inhibitor abolished the vascular smooth muscle cell pERK1/2, the receptor upregulation and normalised CBF and the neurology score despite administration of the inhibitor as late as at 6 h after the start of the SAH. If the raf inhibitor was given 12 h after initiating the SAH there were no significant changes in CBF, neurology score, contractile receptor upregulation and protein levels. There was, however one exception, the protein level for ET_B _and the mRNA levels were depressed also when the drug was given 12 h after the SAH.

A number of mechanisms and receptors have been proposed to account for the late cerebral ischemia that occurs after SAH with subsequent high morbidity. Here we show that by intracisternal administration of a specific raf inhibitor this response can be modified which implicates that cerebrovascular smooth muscle receptor upregulation is an important part in the response to SAH. The immunohistochemistry revealed that SAH results in enhanced phosphorylation of pERK1/2 in the smooth muscle cells and that this expression is normalized by SB386023-b treatment. This confirms that specific inhibition of the ras/raf/MEK/ERK1/2 signaling pathway in the cerebrovascular system is associated with the receptor protein expression.

Considerable efforts have been spent at the development and testing of drugs that may antagonize putative spasmogens, but to this date no effective drug exists [1,6,25]. The latest in this line is the ET receptor antagonist clazosentan [26]; the first preliminary study revealed an effect on large artery vasospasm but had no effect on the neurology deficit. The clinical trials with the selective ET_A _antagonist clazosentan demonstrated that clazosentan reduces the severity of vasospasm following aneurysmal SAH; however, there was no positive effect in the outcome of the patients. This supports our view that the inhibition of only one receptor system will not remedy other receptor systems involved. Instead, the mechanism responsible for the receptor upregulation might be a more promising target.

Since the etiology of cerebral vasospasm is multifactorial, we hypothesize that several receptors are involved in the development and maintenance of this prolonged pathological contraction [27]. Our studies have demonstrated involvement of at least three groups of contractile cerebrovascular receptors in experimental SAH [15,24,27-30] and in human stroke [31]; this alludes to the possibility of the involvement of several receptor systems in late cerebral ischemia and makes it attractive to search for a key signal-transduction mechanism involved in the upregulation process. We observed that SAH results in receptor upregulation not only of the large cerebral arteries but as shown in Figure 6 also of vascular smooth muscle cell receptors in brain microvessels. This latter observation might be of clinical relevance since the clazosentan study (37) and the early nimodipine study [32] revealed partial reversal of angiographic vasospasm but no or minor effect on clinical outcome.

Targeting only one of several important subtypes of receptors such as those of endothelin-1, serotonin or angiotensin II separately in clinical or experimental trials might prevent cerebral ischemia to a certain degree as seen in the literature, but treatments aimed at a common signaling pathway would be more beneficial since further possible receptors and inflammatory mechanisms might be involved. In addition, the different receptor antagonists have profound systemic vascular effects which make their specific effects on the cerebral circulation difficult to obtain. We have demonstrated that upregulation of several of the contractile receptors in the cerebral vasculature are interconnected by their signal transduction pathways [19,28,29,33,34]. Hence, blocking common signal transduction pathways can simultaneously affect the signaling for production of these receptor subtypes [35]. Cerebral ischemia elicits a wide range of responses resulting in activation of a number of intracellular pathways. In particular there is an involvement of the mitogen-activated protein kinases (MAPK) signalling pathway in cerebral vasospasm [36-38].

The MAPK is a family of serine/threonine protein kinases involved in cellular differentiation, proliferation and survival [39,40]. Interestingly it is only the ERK1/2 pathway and not those of p38 or JNK that is active during the first 24 h after experimental SAH [41]. JNK and p38 are only activated at 48 h and this may relate to inflammation and apoptosis which occur later in the process. In addition, SB386023-b is selective for the ERK1/2 pathway since it did not inhibit the JNK and p-38 MAPK pathways [41]. Several other studies have evaluated the effect of available raf inhibitors on cerebrovascular G-protein coupled receptors [19,42,43]. In additional the JNK, p38 and PKC inhibitors have been tested. However the ERK1/2 seems to be more important for the receptor upregulation [44,45]. The raf inhibitor SB386023-b was the one showing the best inhibiting effect on the cerebrovascular receptors and the most specific for the MAPK pathway, which is the reason why this inhibitor was chosen. In the present study we have performed a study closely related to the clinical reality. Thus, SB386023-b was found to have no acute effect on the CBF, ICP or on the tone of the MCA or on its contractility.

It is notable that the effect of the raf inhibitor was equally strong when administration was started 6 h after the SAH as when given at the time of the SAH (starting at time 0 h), but had no significant effect when it was given 12 h after the SAH. The present study was designed to examine the possibility of a therapeutic window of relevance to the clinic. Interestingly, we observed that the upregulation of receptors are located to the cerebral blood vessels SMC (both large arteries and micro vessels), with no signs of upregulation of either receptors or activation of pERK1/2 in the adjacent brain tissue. This is important since only a fraction of the SAH patients have angiographic vasospasm [46]. In addition, we observed that both the large cerebral arteries belonging to the circle of Willis and the cerebral micro vessels in the brain parenchyma are involved to the same extent in cerebral ischemia after SAH. The raf inhibitor SB386023-b affects all vessel types. One possibility may be that the micro vessels are involved in the ischemia that occurs without angiographic vasospasm and the larger arteries might be involved in ischemia where vasospasm takes place or can be visualized angiographically.

## Conclusion

In conclusion, we have provided two important observations: First blockade of pERK1/2 with a raf inhibitor in the cerebrovascular smooth muscle cells prevents the upregulation of contractile receptors and the associated reduction in the regional CBF and neurology score after SAH. Second the two phenomena are associated and putatively treatable also in the clinical setting since administration of the raf inhibitor first applied 6 h after the induction of the SAH showed to have effect. Therefore, we suggest that this is a novel target for treatment of cerebrovascular disorders such as cerebral ischemia after SAH.

## Methods

All animal procedures were carried out strictly within national laws and guidelines and approved by the Danish Animal Experimentation Inspectorate and the Ethical Committee for Laboratory Animal Experiments at the University of Lund.

### Rat subarachnoid hemorrhage model

Subarachnoid hemorrhage was induced by a model originally devised by Svendgaard et al [47] and carefully described by Prunell et al [48]. Svendgaard has in an elegant series of studies carefully analysed the correlation between amount of blood, angiographic vasoconstriction, CBF and cerebral metabolism. In a previous study using the same SAH model Delgado et al [49] have revealed a biphasic vasospasm in angiographic examinations of the arteries with a maximal acute vasoconstriction at ten minutes and a late maximal constriction at two days after SAH.

Male Sprague-Dawley rats (350-400 g) were anaesthetized using 5% halothane (Halocarbon Laboratories, River Edge, New Jersey) in N_2_O/O_2 _(30:70). The rat was intubated and artificially ventilated with inhalation of 0.5-1.5% halothane in N_2_O/O_2 _(70:30) during the surgical procedure. The depth of anaesthesia was carefully monitored and the respiration checked by regularly withdrawing arterial blood samples for blood gas analysis (Radiometer, Copenhagen, Denmark). An electric temperature probe was inserted into the rectum of the rat to record the temperature, and found to be maintained at 37°C. An arterial catheter was placed in the tail artery to measure blood pressure and a catheter to monitor intracranial pressure (ICP) was placed in the subarachnoid space under the subocciptal membrane. At either side of the midline of the skull, 3 mm from the midline and 4 mm anteriorly from the bregma, holes were drilled through the skull bone down to the dura mater (without perforation) allowing the placement of two laser-Doppler flow probes to measure cortical CBF. Finally, a 27 G blunt canula with side hole was introduced 6.5 mm anterior to bregma in the midline at an angle of 30° to the vertical. With the aperture pointing to the right, the needle was lowered until the tip reached the skull base 2 to 3 mm anterior to the chiasma. After 30 minutes of equilibration 250 μl of blood was withdrawn from the tail catheter and injected intracranially via this canula at a pressure equal to the mean arterial blood pressure (MABP) (80-100 mmHg). Subsequently the rat was kept under anaesthesia for another 60 minutes to allow recovery from the cerebral insult after which catheters were removed and incisions closed. The rat was then revitalized and extubated. A subcutaneous injection of carprofen (4.0 mg/kg) (Pfizer, Denmark) was administered and the rat was hydrated subcutaneously using 40 ml isotonic sodium chloride at the end of the operation and at day one. During the period, the rat was monitored regularly, and if showing severe distress the animal was prematurely killed. In addition, a series of sham-operated rats were prepared. Two types of sham animals were studied; no fluid injection or injection of saline (250 μl) during 15 min to avoid any change in ICP [50]. Since both procedures revealed the same outcome, they were grouped together in the statistical analysis. After two days either autoradiography measurements or harvesting of vessels were done (see below for details)

### Neurology scoring

All surviving animals were neurologically examined using an established scoring system of 0 -5 (Table 1) [21,22]. The animals were tested on the day before surgery. On day 1 and 2 after surgery each animal was tested twice. All animals were graded by personnel blinded to the experimental groups of the animals, and subjectivity in the observations was reduced by the involvement of two observers in the testing of each animal.

### Measurement of the effect of the raf inhibitor on the cortical CBF and ICP

This group of animals went through the same procedure as the above-mentioned SAH animals until the injection of blood. To investigate the effect of the raf inhibitor on the cortical CBF and ICP 20 μl; 10^-6 ^M of SB386023-b (a kind gift from Dr A A Parsons, GSK, UK) was given at the time point 0 h and 6 h after the induced SAH. The SB386023-b was injected intracisternally via the occipital membrane into the cisterna magna. The cortical CBF and ICP were measured during the entire time period 0-7 h after the SAH. After the experiment the animals were decapitated. Control sham animals received the same volume of saline.

### Rat subarachnoid hemorrhage model with raf inhibition

This group of animals went through the same procedure as the above-mentioned SAH animals. In addition they were treated with 20 μl; 10^-6 ^M of SB386023-b or the same volume of vehicle. Three groups of treated animals were examined; (i) 20 μl; 10^-6 ^M SB386023-b was repeatedly injected intracisternally at 0, 6, 12, 24 and 36 h after the induced SAH (ii) 20 μl; 10^-6 ^M SB386023-b was repeatedly injected intracisternally at 6, 12, 24 and 36 h after the induced SAH or (iii) 20 μl; 10^-6 ^M SB386023-b was repeatedly injected intracisternally in the cistern magna at 12, 24 and 36 h after the induced SAH. The dose was chosen on the basis of previous detailed work on isolated arteries [19] and *in vivo *study with SAH and ERK1/2 inhibition [28]. The dose used was chosen at near maximum inhibition and calculation of cerebrospinal fluid volume/turn over.

### Autoradiographic measurements of regional CBF

Regional and global cerebral blood flow was measured by a model originally described by Sakurada [51] and modified by Gjedde [52].

In brief, after 48 hours of observation rats in the various groups (sham, SAH, SAH + vehicle and SAH treated with the raf inhibitor) were anaesthetized using 5% halothane in N_2_O/O_2 _(30:70). The animal was intubated and artificially ventilated with inhalation of 0.5 -1.5% halothane in N_2_O/O_2 _(70:30) during the surgical procedure. The anaesthesia and the respiration were monitored by regularly withdrawing arterial blood samples for blood gas analysis (Radiometer AS, Denmark). A catheter to measure MABP was placed in the right femoral artery and a catheter for blood sampling was placed in the left femoral artery. This catheter was connected to a constant velocity withdrawal pump (Harvard apparatus 22, USA) for mechanical integration of tracer concentration. In addition, a catheter was inserted in one femoral vein for injection of heparin and for infusion of the radioactive tracer. The MABP was continuously monitored with a Powerlab Unit (ADInstruments, UK). A temperature probe was inserted into the rectum of the rat to record the temperature, which was regularly maintained at 37°C. The hematocrit was measured by a hematocrit centrifuge (Beckman Microfuge 11, USA). After 30 minutes of equilibration a bolus injection of 50 uCi of ^14^C-iodoantipyrine 4[N-methyl-^14^C] (Perkin-Elmer, Boston, USA) was given i.v. Arterial blood (122 μl) was withdrawn over 20 seconds. Immediately after this the animal was decapitated, the brain removed and immersed in isopentane chilled to - 50°C. The arterial blood sample was transferred to liquid scintillation counting vials containing 1 ml mixture of Soluene-350 and Isopropanol (1:1). The β-radioactivity scintillation counting was performed on the samples with a program that included quench correction (Packard 2000 CA, Denmark). The ^14^C activity in the tissue was determined after sectioning the brain in 20 μm sections at -20°C in a cryostat (Wild Leitz A/S, Glostrup, Denmark). The sections were exposed to x-ray films (Kodak, Denmark) together with ^14^C methylmethacrylate standards (Amersham Life Science, England) and exposed the films for 20 days. Densities of the autoradiograms were measured with a Macintosh computer equipped with an analog CF 4/1 camera (Kaiser, Germany) and a transparency flat viewer (Color-Control 5000, Weilheim, Germany). The ^14^C content was determined in several brain regions (see Table 2). The CBF was calculated from the brain tissue ^14^C activity determined by autoradiography using Gjedde et al.'s equation [52].

### Harvest of cerebral arteries

After 48 hours of observation sham, SAH treated with SB386023-b or SAH + vehicle operated rats (see above SAH model) were anaesthetized with CO_2 _and decapitated. The brains were quickly removed and chilled in ice-cold bicarbonate buffer solution. Under a dissection microscope, the middle cerebral artery (MCA), the basilar artery (BA) and circle of Willis were dissected out. The MCA and BA were immediately mounted in myographs for in vitro pharmacology or snap frozen at -80°C and examined by real-time PCR or immunohistochemistry.

### In vitro pharmacology myograph experiments

For contractile experiments a sensitive myograph was used for recording the isometric tension in isolated cerebral arteries [53,54]. The vessels were cut into 1 mm long cylindrical segments and mounted on two 40 μm in diameter stainless steel wires in a Myograph (Danish Myo Technology A/S, Denmark). One wire was connected to a force displacement transducer attached to an analog-digital converter unit (ADInstruments, Oxford, UK). The other wire was connected to a micrometer screw, allowing fine adjustments of vascular tone by varying the distance between the wires. Measurements were recorded on a computer by use of a PowerLab unit (ADInstruments). The segments were immersed in a temperature controlled buffer solution (37°C) [50]. The vessels were stretched to an initial resting tone of 2 mN and then allowed to stabilize at this tone for 1 hour. The contractile capacity was determined by exposing the vessels to an isotonic solution containing 63.5 mM of K^+^, obtained by partial change of NaCl for KCl in the above buffer. The contraction induced by K^+ ^was used as reference for the contractile capacity [54]. Only vessels responding by contraction of at least 2.0 mN to potassium for BA and 0.8 mN to potassium for MCA were included in the study. The presence of the endothelium was checked by precontracting the vessel using 5-HT (10 ^-6.5 ^M) (Sigma, St Louis, USA) and subsequently exposing the segments to carbachol (10^-5 ^M) (Sigma, St Louis, USA). A relaxant response of the precontracted tension was considered indicative of a functional endothelium [24].

Concentration-response curves were obtained by cumulative application of 5-CT (Sigma, St. Louis, USA) in the concentration range 10 ^-12 ^to 10 ^-5 ^M, ET-1 (AnaSpec, San Jose, USA) in the concentration range 10 ^-14 ^to 10 ^-7 ^M, SB386023-b (a kind gift from Dr A A Parsons, GSK, UK) in the concentration range 10 ^-12 ^to 10 ^-6 ^M and Ang II (Sigma, St. Louis, USA) in the concentration range 10 ^-12 ^to 10 ^-6 ^M. Before application of Ang II the arteries were pretreated with the AT_2 _receptor antagonist PD123319 (10 ^-5.5 ^M) for 30 minutes (Sigma, St. Louis, USA). The concentration-response curves for SB386023-b were investigated both with and without precontraction with 5-HT (10 ^-6.5 ^M) (Sigma, St Louis, USA).

### RNA isolation

To quantify mRNA for the ET_A_, ET_B_, AT_1_, AT_2 _and 5-HT_1B _receptors, RT-PCR and real-time detection monitoring the PCR products was employed.

Total cellular RNA was extracted from BA, MCA and circle of Willis using the Trizol RNA isolation kit (Invitrogen, USA) following the suppliers instructions. Briefly, the arteries were homogenized in 1 ml of Trizol (Invitrogen, Sweden) by using a TissueLyser (VWR, Sweden). Subsequently 200 μl of chloroform was added and the samples were incubated in room temperature for 3 min, followed by centrifugation at 15000 g for 15 min at 4°C. The supernatant was collected and the organic phase discarded. 200 μl of chloroform was again added to remove all traces of phenol and the samples were centrifuged at 15000 g for 15 at 4°C. The aqueous supernatant was again collected and to precipitate the RNA equal amount of isopropanol was added and the samples incubated overnight at -20°C.

Subsequently, the RNA was centrifuged at 15000 g for 20 min at 4°C. The supernatant was discarded and the resulting pellet was washed with 75% ethanol, air dried and re-dissolved in diethylpyrocarbonate treated water. Total RNA was determined using a GeneQuant Pro spectrophotometer measuring absorbance at 260/280 (Amersham Pharmacia Biotech, Uppsala, Sweden).

### Real-time PCR

Reverse transcription of total RNA to cDNA was carried out using the Gene Amp RNA kit (Perkin-Elmer Applied Biosystems, USA) in a Perkin-Elmer 2400 PCR machine at 42°C for 90 min and then 72°C for 10 min. The real-time quantitative PCR was performed with the GeneAmp SYBR Green PCR kit (PE Applied Biosystems) in a Perkin-Elmer real-time PCR machine (GeneAmp 5700 sequence detection system). The above synthesized cDNA was used as a template in a 25 μl reaction volume and a no template was included in all experiments. The system automatically monitors the binding of a fluorescent dye to double-strand DNA by real-time detection of the fluorescence during each cycle of PCR amplification. Specific primers for the rat ET_A_, ET_B_, AT_1_, AT_2 _and 5-HT_1B _receptor and house keeping gene elongation factor-1 (EF-1) were designed by using the Primer Express 2.0 software (PE Applied Biosystems) and synthesized by TAG Copenhagen A/S (Copenhagen, Denmark). For the primer sequence, refer to our previous studies [27].

The housekeeping gene EF-1 is used as a reference, since it is continuously expressed to a constant amount in cells.

The PCR reaction was carried out as follows: 50°C for 2 min, 95°C for 10 min and the following 40 PCR cycles with 95°C for 15 sec and 60°C for one min. Each sample was examined in duplicates. To verify that each primer-pair only generated one PCR product at the expected size a dissociation analysis was performed after each real-time PCR run. A blank control (without template) was used in all experiments. To prove that the cDNA of EF-1 and the ET, AT and 5-HT_1B _receptors were amplified with a similar efficacy during real-time PCR, a standard curve were made.

### Tissue Lysis and Protein Content Determination

After dissection of the circle of Willis arteries, the vessels were collected and placed on ice, homogenized in lysis-buffer with protease- and phosphatase inhibitors. After 20 min incubation in lysis buffer on ice, homogenates were centrifuged at 4500 g for 10 min at 4°C and supernatant collected. Total protein concentration was determined using a BioRad DC kit (Hercules, CA, USA) and measuring absorbance at 750 nm on a Genesys 10 spectrophotometer (Thermo, Waltham, MA, U.S.A.). Lysates were used immediately or stored at -80°C.

### Western Blot Analysis

Proteins of interest were evaluated in circle of Willis arteries from the various groups.

Lysates were dissolved in Tris-glycine SDS sample buffer and boiled for 5 min. Equal amounts of protein (50 μg/lane) were loaded on a 8% Tris-glycine gel (Invitrogen A/S, Taastrup, Denmark) and separated by SDS-PAGE. Molecular weight markers (New England BioLabs, Ipswich, MA, USA) were loaded on each gel for protein band identification. After separation, proteins were transferred to a nitrocelullose membrane (BioRad, Hercules, CA, USA). Subsequently the membrane was blocked with 6.5% non-fat milk in Tween-TBS (T-TBS) overnight 4°C. Membranes were then incubated with the primary antibody of interest: pERK1/2 (1:5000 dilution; Promega, Madison, WI, U.S.A.) or β-actin (1:1000 dilution; Sigma, Saint Louis, USA) for 1 h at 37°C, followed by 3 × 5 min wash with T-TBS. Subsequently the membranes were incubated with the appropriate secondary antibody: goat anti-rabbit IgG-horseradish peroxidase or goat anti-mouse IgG-horseradish peroxidase (1:5000; Pierce, Rockford, IL, U.S.A) for 1 h at room temperature, followed by 5 × 5 min wash with T-TBS. Levels of β-actin were used to confirm equal loading of the lanes. The membranes were developed using the Supersignal Dura kit (Pierce, Rockford, IL, U.S.A.) and visualized using a Fujifilm LAS-1000 Luminiscent Image Analyzer (Stamford, CT, U.S.A.).

#### Immunohistochemistry

For immunohistochemistry the indirect immunofluorescence method was used. The BA, with surrounding brain tissue were dissected out and frozen in ice cold isopentane. They were then sectioned into 10 μm thick slices in a cryostat. The cerebral artery crysections were fixed for 10 minutes in ice cold acetone and thereafter rehydrated in phosphate buffer solution (PBS) containing 0.25% Triton X-100 for 15 minutes. The tissue was then permeabilized and blocked for 1 hour in blocking solution containing PBS, 0.25% Triton X-100, 1% BSA and 5% normal donkey serum. The sections were incubated over night at 4°C with the following primary antibodies: rabbit antihuman ET_B _(IBL, 16207), diluted 1:400, goat anti mouse 5-HT_1B _(Santa Cruz Biotechnologies, sc-1461), diluted 1:100, AT_1 _(Santa Cruz Biotechnologies), diluted 1:100, mouse anti rat CD31 (Serotec, MCA1746), diluted 1:200, rabbit antiphospho ERK 1/2 MAPK (Cellsignalling #4376) diluted 1:50. and mouse anti rat smooth muscle actin (Serotec, MCA1905T) diluted 1:100. All dilutions were done in PBS containing 0.2% Triton X- 100, BSA 1% and 2% normal donkey serum. Sections were subsequently washed with PBS and incubated with secondary antibody for 1 hour at room temperature. The secondary antibody used were donkeyantimouse Cy™5 conjugated (JacksonImmunoResearch, 715-175-150), donkeyantirabbit Cy™3 conjugated (JacksonImmunoResearch, 711-165-152) diluted 1:200 in PBS containing 0.2% TritonX- 100 and BSA 1%. The sections were washed subsequently with PBS and mounted with permafloure mounting medium (Beckman coulter, PN IM0752). The same procedure was used for the negative controls but primary antibodies were omitted. The immunoreactivity of the antibodies were visualized and photographed with a Nikon Eclipse E800 microscope fitted with fluorescence optics at the appropriated wavelength.

#### Calculations and statistics

Data are expressed as mean ± standard error of the mean (s.e.m.), and n refers to the number of rats. Statistical analyses were performed with Kruskal-Wallis non-parametric test with Dunn's post-hoc test, where P < 0.05 was considered significant.

##### In vitro Pharmacology

Contractile responses in each segment are expressed as percentage of the 63.5 mM K^+ ^induced contraction. E_max _value represents the maximum contractile response elicited by an agonist and the pEC_50 _the negative logarithm of the drug concentration that elicited half the maximum response. For biphasic responses, E_max(1) _and pEC_50(1) _describes the high affinity phase and E_max(2) _and pEC_50(2) _describes the low affinity phase.

##### Real-time PCR

Data were analysed with the comparative cycle threshold (CT) method [30]. The CT values of EF-1 mRNA were used as a reference to quantify the relative amount of ET_A_, ET_B_, AT_1_, AT_2 _and 5-HT_1B _mRNA. The relative amount of mRNA was calculated with the CT values of ET_A_, ET_B_, AT_1_, AT_2 _and 5-HT_1B _receptor mRNA in relation to the CT values of EF-1 mRNA in the sample by the formula *X*_0_/*R*_0 _= 2*^CtR-CtX^*, where *X*_0 _is the original amount of target mRNA, *R*_0 _is the original amount of EF-1 mRNA, *CtR *is the *C_T _*value for EF-1 and *C_T_X *is the *C_T _*value for the target.

##### Western Blot

Cerebrovascular protein lysates from the different groups were compared. Cerebral arteries from 2 animals were pooled for each group of experiment and each experiment was repeated 3 times. Quantitation of band density was performed with the electrophoresis computer analysis program Fujifilm Science Laboratory Image Gauge 4.0. The immunoblot optical density values were determined with repeated measurement and presented as percentage activity in the treated groups compared with the sham in which the sham group was set to 100%.

##### Immunohistochemistry

The images were analysed using the ImageJsoftware http://rsb.info.nih.gov/ij. The fluorescence in 4-6 different areas in each artery was measured and a mean value was calculated. These values are presented as percentage fluorescence in the SAH groups compared to the sham group, where the sham group is set to 100%.

## Authors' contributions

All authors read and approved the final manuscript. SA participated in the design of the study, performed the main part of the experiments, analyzed the data, and wrote the manuscript. AM performed the immunohistochemisty experiments and participated in writing the manuscript. LE conceived the study, guided the experimental procedures, and participated in writing the manuscript.
